# Supplementary material for: Examination of marketing mix performance in relation to sustainable development of the Poland’s confectionery industry
Source: PLoS One. 2020 Oct 26;15(10):e0240893. doi: 10.1371/journal.pone.0240893 (PMC7588123; doi:10.1371/journal.pone.0240893)
Supplement: S1 Table — (PDF) [file pone.0240893.s001.pdf]

S1 Table. The Questionnaire's Template

No.	Questions	Responses codes
**1.**	The company designs products taking into account their impact on the state of the environment.	0, 1, 2, 3, 4, 5, 6
**2.**	The company consciously saves energy, water and fuels needed for production.	0, 1, 2, 3, 4, 5, 6
**3.**	The company eliminates or limits the emission of harmful gases, dusts, fragrances, industrial wastewater, and production waste.	0, 1, 2, 3, 4, 5, 6
**4.**	The company uses renewable sources.	0, 1, 2, 3, 4, 5, 6
**5.**	The company consciously eliminates or limits the types and quantities of materials used to make packaging, e.g. film, paper, plastic, aluminum, etc.	0, 1, 2, 3, 4, 5, 6
**6.**	The enterprise uses packaging made from raw materials wholly or partly recycled.	0, 1, 2, 3, 4, 5, 6
**7.**	The company makes modifications to create ecological packaging.	0, 1, 2, 3, 4, 5, 6
**8.**	The company designs products taking into account their impact on consumer health.	0, 1, 2, 3, 4, 5, 6
**9.**	The enterprise knowingly eliminates or limits the content of such raw materials as white sugar, chemical preservatives, flavor enhancers, artificial colors, trans fats.	0, 1, 2, 3, 4, 5, 6
**10.**	The company effectively manages human capital while respecting employee rights.	0, 1, 2, 3, 4, 5, 6
**11.**	The company provides its employees with safe and healthy working conditions.	0, 1, 2, 3, 4, 5, 6
**12.**	The company ensures fair remuneration.	0, 1, 2, 3, 4, 5, 6
**13.**	The enterprise includes in the prices of products the costs of purchasing raw materials, energy and materials for production.	0, 1, 2, 3, 4, 5, 6
**14.**	The enterprise includes transport and storage costs in product prices.	0, 1, 2, 3, 4, 5, 6
**15.**	The enterprise includes employee costs in product prices e.g. ensuring health and safety, social security.	0, 1, 2, 3, 4, 5, 6
**16.**	The enterprise includes in the prices of products the costs associated with the elimination or reduction of harmful gases, dusts, fragrances, industrial wastewater and production waste.	0, 1, 2, 3, 4, 5, 6
**17.**	The company includes in the prices of products the costs of recycling packaging from consumed food products.	0, 1, 2, 3, 4, 5, 6
**18.**	The company includes in the prices of products the costs of treatment related to the consumption of food products e.g. costs of treating obesity, diabetes, tooth decay, etc.	0, 1, 2, 3, 4, 5, 6
**19.**	The company uses environmentally friendly means of transport e.g. hybrid and electric, and LPG fueled cars.	0, 1, 2, 3, 4, 5, 6
**20.**	The company uses environmentally friendly resources and devices.	0, 1, 2, 3, 4, 5, 6
**21.**	The company is withdrawing transport with high emissions and noise.	0, 1, 2, 3, 4, 5, 6
**22.**	The company optimizes the delivery of products by choosing the right routes (shorter, faster routes).	0, 1, 2, 3, 4, 5, 6
**23.**	The company combines deliveries to several recipients simultaneously.	0, 1, 2, 3, 4, 5, 6
**24.**	The company adjusts the means of transport to the amount of transported goods.	0, 1, 2, 3, 4, 5, 6
**25.**	The company makes the most of transport space by properly arranging products in means of transport.	0, 1, 2, 3, 4, 5, 6

**26.**	The company monitors and controls the working time of drivers to avoid road accidents.	0, 1, 2, 3, 4, 5, 6
**27.**	The company optimizes distribution channels by reducing intermediaries involved in distribution processes.	0, 1, 2, 3, 4, 5, 6
**28.**	The company selects intermediaries in terms of ecological and social sensitivity.	0, 1, 2, 3, 4, 5, 6
**29.**	The content of promotional messages is characterized by transparency and credibility.	0, 1, 2, 3, 4, 5, 6
**30.**	The content of promotional messages is educational.	0, 1, 2, 3, 4, 5, 6
**31.**	The content of promotional messages emphasize the pro-ecological and pro-social features of the products.	0, 1, 2, 3, 4, 5, 6
**32.**	The content of promotional messages inform consumers about the company's ecological and social activity.	0, 1, 2, 3, 4, 5, 6
**33.**	The content of promotional messages encourages the purchase of pro-ecological and pro-social products manufactured by the company.	0, 1, 2, 3, 4, 5, 6

Responses options with codes:
(0)-definitely no, (1)-no, (2)-rather no, (3)-not applicable,
(4)-rather yes, (5)-yes, (6)-definitely yes

PARTICULARS

Legal form of the business	☐ Public limited company<br>☐ Limited liability company<br>☐ Limited joint-stock partnership<br>☐ Limited partnership<br>☐ Limited liability partnership<br>☐ General partnership<br>☐ Civil law partnership<br>☐ Sole proprietorship
Number of employees	
Name of the province where the business operates	☐ Lower Silesia<br>☐ Kuyavian-Pomeranian<br>☐ Lublin<br>☐ Lubusz<br>☐ Lodz<br>☐ Lesser Poland<br>☐ Mazovian<br>☐ Opole<br>☐ Subcarpathian<br>☐ Podlasie<br>☐ Pomeranian<br>☐ Silesian<br>☐ Holy Cross<br>☐ Varmia-Mazuria<br>☐ Greater Poland<br>☐ West-Pomeranian
The company's representative	
Professional position	☐ Managerial<br>☐ Non managerial
