# Supplementary material for: Examination of marketing mix performance in relation to sustainable development of the Poland’s confectionery industry
Source: PLoS One. 2020 Oct 26;15(10):e0240893. doi: 10.1371/journal.pone.0240893 (PMC7588123; doi:10.1371/journal.pone.0240893)
Supplement: S2 Table — Qi denotes question’s number which relates to question numbers in S1 Table that also contains their essence. (PDF) [file pone.0240893.s002.pdf]

S2 Table. **Database Q_1-Q_{12}**

**No.**	Q_1	Q_2	Q_3	Q_4	Q_5	Q_6	Q_7	Q_8	Q_9	Q_{10}	Q_{11}	Q_{12}
**1**	2	4	4	5	2	4	1	1	2	5	5	4
**2**	4	5	4	3	4	3	3	3	4	4	5	4
**3**	2	2	4	1	1	2	1	0	0	3	4	4
**4**	3	4	4	4	3	4	3	3	2	4	5	4
**5**	4	6	6	6	6	6	6	2	3	6	6	6
**6**	4	6	6	5	3	6	4	3	3	5	6	6
**7**	6	6	5	5	3	6	4	4	2	5	6	4
**8**	2	2	4	4	0	4	2	1	0	4	6	4
**9**	2	4	4	1	0	1	1	0	0	4	5	2
**10**	2	2	4	2	0	1	1	0	0	3	4	2
**11**	1	2	2	2	0	2	2	0	0	4	4	4
**12**	2	4	4	4	0	2	1	0	0	4	5	4
**13**	5	5	5	5	0	5	4	2	1	5	6	5
**14**	2	2	4	4	0	5	0	0	0	4	5	4
**15**	2	4	5	4	0	4	1	0	0	4	6	6
**16**	1	3	4	4	1	2	2	2	1	4	5	4
**17**	4	2	5	2	0	2	2	1	1	4	6	4
**18**	5	6	4	5	3	6	6	6	6	5	5	5
**19**	2	1	2	1	1	2	2	1	1	4	5	5
**20**	2	2	4	1	0	1	1	0	0	4	5	4
**21**	2	4	4	2	0	2	1	1	1	4	5	4
**22**	4	5	5	5	1	5	4	5	4	6	6	6
**23**	4	5	5	5	1	5	4	4	1	5	5	5
**24**	5	5	5	5	2	5	4	4	1	5	6	5
**25**	4	4	5	4	0	1	1	1	1	4	4	4
**26**	4	4	5	4	0	2	2	1	1	5	5	5
**27**	4	5	5	5	1	2	2	1	1	5	5	5
**28**	4	5	5	5	2	5	2	2	1	5	5	5
**29**	5	5	5	5	2	5	5	4	2	6	6	5
**30**	5	5	5	5	1	4	2	2	1	5	5	4
**31**	5	5	5	5	1	5	2	1	1	5	5	5
**32**	2	4	5	2	1	2	1	1	1	4	5	4
**33**	2	4	5	4	0	2	1	1	1	4	5	4
**34**	4	4	5	5	1	2	1	1	1	5	5	4
**35**	5	5	5	5	2	5	2	2	0	5	5	5
**36**	5	5	5	5	1	5	1	1	1	5	5	5
**37**	1	1	1	1	0	1	1	1	1	4	5	1
**38**	2	5	5	4	1	2	1	1	1	5	5	4

**39**	0	0	0	0	0	0	0	0	0	1	4	0
**40**	6	6	6	5	2	6	5	4	2	5	5	4
**41**	5	5	5	5	1	4	1	1	1	5	6	4
**42**	5	5	5	4	1	5	1	4	1	5	6	4
**43**	4	5	5	4	2	4	2	1	1	5	5	4
**44**	1	1	2	1	1	1	1	1	1	4	5	4
**45**	5	5	5	5	2	4	2	2	1	5	6	4
**46**	4	5	5	4	1	4	1	1	1	5	6	4
**47**	5	5	5	5	2	5	1	1	1	5	5	4
**48**	5	5	5	5	2	4	1	2	2	5	6	4
**49**	4	5	5	4	2	5	1	5	1	5	5	4
**50**	1	4	4	2	1	2	1	0	0	5	5	4
**51**	2	5	4	2	0	2	0	1	0	4	5	4
**52**	1	4	5	1	1	1	0	0	0	4	4	2
**53**	4	5	5	5	2	5	2	2	4	5	5	4
**54**	4	5	4	4	1	4	1	1	1	4	5	4
**55**	4	5	5	5	2	5	4	2	1	4	5	4
**56**	4	5	5	5	1	4	0	1	0	4	6	4
**57**	5	5	5	4	1	4	1	2	2	4	5	4
**58**	1	4	4	1	5	4	1	2	0	4	5	2
**59**	1	1	4	1	1	1	1	1	0	4	4	0
**60**	4	5	5	4	2	4	2	2	1	5	6	4
**61**	4	5	5	4	2	5	2	5	2	5	5	4
**62**	5	5	5	2	1	4	1	1	1	5	5	5
**63**	1	1	1	1	0	1	1	1	1	4	4	1
**64**	1	4	4	1	1	2	1	2	0	4	4	2
**65**	4	5	6	4	1	4	2	2	1	5	5	4
**66**	2	4	4	1	1	1	1	1	0	4	5	1
**67**	4	5	5	5	2	4	1	4	0	5	5	4
**68**	1	4	4	2	1	1	1	2	1	4	4	0
**69**	0	0	0	0	5	0	5	6	5	0	0	0
**70**	4	5	5	5	1	5	5	4	2	5	5	2
**71**	4	5	5	5	1	5	4	4	1	5	5	4
**72**	4	5	5	5	1	4	4	2	1	1	5	4
**73**	4	4	5	5	0	5	4	2	0	4	5	2
**74**	5	6	5	5	2	5	5	4	1	5	6	4
