# Supplementary material for: Examination of marketing mix performance in relation to sustainable development of the Poland’s confectionery industry
Source: PLoS One. 2020 Oct 26;15(10):e0240893. doi: 10.1371/journal.pone.0240893 (PMC7588123; doi:10.1371/journal.pone.0240893)
Supplement: S3 Table — Qi denotes question’s number which relates to question numbers in S1 Table that also contains their essence. (PDF) [file pone.0240893.s003.pdf]

S3 Table. **Database Q_{13}-Q_{18} & Q_{29}-Q_{33}**

No.	Q_{13}	Q_{14}	Q_{15}	Q_{16}	Q_{17}	Q_{18}	Q_{29}	Q_{30}	Q_{31}	Q_{32}	Q_{33}
**1**	5	6	6	2	1	0	4	4	1	2	2
**2**	6	5	4	4	4	3	4	3	3	3	3
**3**	5	5	2	1	0	0	1	1	0	0	0
**4**	6	5	3	2	2	0	4	2	2	1	1
**5**	6	6	6	2	1	0	6	6	4	5	4
**6**	6	6	4	4	6	2	5	3	2	0	0
**7**	6	6	5	4	5	0	3	1	2	1	2
**8**	6	6	4	0	0	0	4	1	0	0	0
**9**	6	6	5	1	0	0	1	1	0	0	0
**10**	5	5	2	1	0	0	1	0	0	0	0
**11**	6	6	5	0	0	0	5	1	0	0	0
**12**	6	5	4	0	0	0	1	1	1	0	0
**13**	6	6	5	1	1	0	3	1	2	2	1
**14**	6	5	0	0	0	0	4	1	0	0	0
**15**	6	5	5	0	0	0	4	1	0	1	0
**16**	5	5	4	2	1	0	3	1	1	0	1
**17**	5	5	4	1	1	0	4	1	0	0	0
**18**	6	4	5	4	4	3	6	4	0	0	0
**19**	5	4	4	1	1	1	4	1	1	1	1
**20**	6	5	5	0	0	0	4	1	0	0	0
**21**	5	5	4	1	1	0	4	1	1	1	1
**22**	6	5	5	4	2	1	4	2	1	1	2
**23**	6	5	5	5	4	1	4	1	1	1	1
**24**	6	5	5	5	2	0	4	1	1	1	1
**25**	5	5	4	1	1	0	4	1	1	1	0
**26**	5	5	2	0	1	0	4	1	1	1	1
**27**	5	5	4	1	1	0	4	1	1	1	1
**28**	5	5	4	1	1	0	4	2	1	1	1
**29**	6	6	5	2	2	1	5	1	1	1	1
**30**	5	5	4	1	1	0	5	1	1	1	2
**31**	5	5	4	1	1	0	5	2	1	1	1
**32**	5	5	4	1	1	0	4	1	1	1	1
**33**	6	5	4	1	1	0	4	1	1	0	0
**34**	5	5	4	1	1	1	5	2	1	0	0
**35**	6	6	5	1	1	1	5	2	2	1	2
**36**	6	5	4	1	1	1	5	2	1	0	0
**37**	5	5	1	0	0	0	3	3	3	3	3
**38**	6	5	4	1	1	0	4	1	0	0	0

**39**	4	1	1	0	0	1	2	0	5	5	5
**40**	6	6	5	1	1	1	5	1	1	1	1
**41**	5	5	4	1	1	0	4	0	0	2	2
**42**	5	5	4	1	1	1	4	1	1	2	2
**43**	5	5	4	1	1	0	4	0	4	1	2
**44**	5	4	1	1	1	0	3	3	1	1	1
**45**	6	5	5	1	0	0	5	2	1	1	4
**46**	5	4	4	1	1	0	4	1	1	1	1
**47**	5	5	4	1	1	0	4	1	1	0	0
**48**	6	5	5	1	1	0	5	2	4	4	1
**49**	6	5	4	2	1	0	5	1	1	1	1
**50**	5	5	4	1	1	1	4	1	1	1	1
**51**	6	5	2	1	1	0	4	0	0	0	0
**52**	5	4	2	1	1	0	3	3	3	3	3
**53**	6	5	4	1	1	1	5	1	1	1	4
**54**	5	4	4	2	1	0	4	2	1	0	4
**55**	6	5	4	0	1	0	4	1	1	1	1
**56**	6	5	4	0	1	0	4	1	1	1	1
**57**	6	5	4	0	0	0	4	0	0	0	0
**58**	5	5	4	0	1	0	5	1	1	1	2
**59**	4	1	1	0	0	0	1	1	1	1	1
**60**	6	5	5	1	0	0	5	2	2	1	1
**61**	6	5	5	1	1	1	4	2	1	0	2
**62**	6	5	4	1	1	0	4	0	0	0	0
**63**	5	5	2	1	1	0	1	1	1	1	1
**64**	5	4	2	1	1	0	2	1	0	0	0
**65**	6	5	5	1	1	0	4	1	1	0	4
**66**	5	5	2	1	1	0	4	1	1	1	1
**67**	6	5	5	0	0	0	5	2	1	1	1
**68**	5	4	2	1	1	0	4	1	1	1	4
**69**	4	2	0	6	5	2	6	5	6	6	6
**70**	6	5	4	1	1	0	4	1	2	1	2
**71**	6	5	4	1	1	0	4	1	2	1	2
**72**	6	5	4	1	1	0	4	1	4	1	2
**73**	5	4	4	1	1	0	2	0	1	1	2
**74**	6	5	2	1	1	0	2	0	1	1	1
