# Supplementary material for: Examination of marketing mix performance in relation to sustainable development of the Poland’s confectionery industry
Source: PLoS One. 2020 Oct 26;15(10):e0240893. doi: 10.1371/journal.pone.0240893 (PMC7588123; doi:10.1371/journal.pone.0240893)
Supplement: S4 Table — Qi denotes question’s number which relates to question numbers in S1 Table that also contains their essence. (PDF) [file pone.0240893.s004.pdf]

S4 Table. **Database Q_{19}-Q_{28}**

No.	Q_{19}	Q_{20}	Q_{21}	Q_{22}	Q_{23}	Q_{24}	Q_{25}	Q_{26}	Q_{27}	Q_{28}
**1**	1	4	3	6	6	6	5	4	4	0
**2**	3	3	3	3	5	5	5	3	4	3
**3**	1	1	2	4	5	4	5	3	4	0
**4**	2	2	2	3	4	4	4	4	4	1
**5**	0	3	2	6	6	6	6	6	6	3
**6**	3	4	3	6	4	6	6	4	4	2
**7**	0	2	4	5	5	4	6	6	6	0
**8**	0	2	0	5	6	5	5	6	5	0
**9**	1	1	0	5	5	4	4	5	2	0
**10**	0	0	0	4	4	4	4	2	3	0
**11**	0	2	2	5	5	5	5	5	2	0
**12**	0	1	1	5	5	4	4	5	2	0
**13**	0	1	2	5	5	4	4	6	5	0
**14**	0	2	1	5	5	5	2	5	1	0
**15**	0	1	1	5	6	4	4	6	1	0
**16**	0	1	4	5	5	5	4	4	4	0
**17**	1	1	4	5	5	2	2	5	1	0
**18**	2	2	5	5	6	6	6	3	6	4
**19**	1	1	4	4	4	1	1	4	1	0
**20**	1	0	2	4	5	5	4	5	1	0
**21**	0	1	2	5	4	2	2	5	1	0
**22**	2	4	4	6	6	5	5	6	2	1
**23**	2	5	5	5	5	5	4	6	5	2
**24**	1	4	4	5	5	5	5	5	4	1
**25**	1	1	1	4	5	4	4	5	1	0
**26**	1	1	2	4	5	5	4	4	1	0
**27**	1	2	1	5	5	5	2	5	1	0
**28**	1	2	2	5	5	5	2	5	1	0
**29**	2	5	5	5	5	5	4	5	5	1
**30**	2	5	4	5	5	5	4	5	2	0
**31**	1	4	4	5	5	5	4	5	5	1
**32**	1	2	1	5	5	5	2	4	1	0
**33**	1	2	1	5	6	5	4	5	2	1
**34**	2	2	1	4	5	4	4	4	1	1
**35**	2	5	4	5	5	5	4	5	4	1
**36**	2	5	4	5	5	5	5	5	2	1
**37**	1	1	1	5	5	5	5	4	1	0
**38**	1	1	2	6	6	5	5	4	1	1

**39**	2	5	4	4	2	4	2	4	5	1
**40**	2	5	5	5	5	5	5	5	4	1
**41**	1	4	4	5	5	5	4	4	4	1
**42**	1	2	2	5	5	4	4	4	4	0
**43**	0	4	1	6	6	4	4	5	2	0
**44**	1	1	1	4	4	4	4	4	1	1
**45**	1	4	4	6	6	5	4	5	4	0
**46**	1	2	1	5	5	4	4	4	1	0
**47**	1	1	1	5	5	4	2	4	4	1
**48**	1	4	4	5	5	5	5	5	4	1
**49**	1	5	5	6	5	5	5	5	5	0
**50**	1	4	4	5	5	5	2	4	4	1
**51**	1	4	4	5	5	5	5	5	5	0
**52**	1	1	1	4	5	5	5	4	1	0
**53**	2	4	4	5	5	5	4	4	4	1
**54**	2	2	1	5	5	4	4	2	4	2
**55**	1	2	2	5	5	5	4	3	2	1
**56**	2	2	2	4	5	4	4	4	2	0
**57**	1	2	1	4	5	5	4	4	1	0
**58**	1	1	1	5	5	5	4	5	1	0
**59**	0	0	1	4	4	4	4	0	0	0
**60**	2	5	4	5	5	5	5	4	4	1
**61**	1	1	1	5	5	5	4	5	1	1
**62**	1	2	1	5	6	6	4	5	2	0
**63**	1	1	1	5	4	4	4	2	1	1
**64**	1	1	1	5	5	4	4	4	1	1
**65**	1	4	4	5	6	5	2	5	4	0
**66**	1	1	1	4	5	4	4	2	1	0
**67**	1	4	4	5	5	5	1	5	4	1
**68**	1	1	1	4	4	4	2	4	1	0
**69**	4	6	6	6	2	2	2	1	5	1
**70**	2	4	4	6	5	5	5	4	5	0
**71**	2	4	5	6	5	5	5	4	5	1
**72**	2	4	4	5	6	5	5	4	5	0
**73**	2	4	4	6	6	4	1	5	4	1
**74**	1	5	5	5	5	5	1	4	4	1
