# Supplementary material for: Examination of marketing mix performance in relation to sustainable development of the Poland’s confectionery industry
Source: PLoS One. 2020 Oct 26;15(10):e0240893. doi: 10.1371/journal.pone.0240893 (PMC7588123; doi:10.1371/journal.pone.0240893)
Supplement: Attachment — Submitted filename: ResponseToReviewers.pdf [file pone.0240893.s005.pdf]

Pawel Tadeusz KAZIBUDZKI

Opole University of Technology,
Faculty of Economics and Management,
St. Luboszycka 7, 45-036 Opole, Poland;
ORCID: 0000-0003-1203-4235
p.kazibudzki@po.edu.pl

September 30, 2020

Editorial Board of the Journal – PLOS ONE

Responses to the Reviewers

Dear Professors – the order of our answers for your reviews was decided to be as in the editorial system of PLOS ONE and the email we received from the Editorial Office. As we are unaware of your identities we will call you Reviewer No. 1 and Reviewer No. 2. The numbers standing next to your anonymous identity are assigned for the order of your appearance in the editorial system of the Journal and our responses identification and have no other meaning whatsoever.

Reviewer No. 1

Dear Reviewer – we would like to thank you for your time and appreciation of our research efforts. We owe you so much for your exceptionally positive judgment of our examination. Obviously, we fully fulfilled your suggestion to add some references in Section 2.2 of the research paper. They are introduced as positions 37-39 and 40-46. Thank you so much for your benevolence!

Reviewer No. 2

Dear Reviewer – we would like to thank you for your time and appreciation of our research efforts. We truly appreciate your very positive judgment of the paper. Once we saw your suggestion to improve the survey we thought we wished we had known it before we have done our research. At the present moment the survey is done and, for a few reasons but mostly financial, there is no way of its complete iteration. The problem seemed to be a dead end – but, at the end, it presented itself as the opportunity. Now, we know that our research benefited better in the way the suggestion was obtained i.e. after our examination was completed. We will explain it now.

The paramount reason why we applied in our research the methodology which enables quantification of qualitative attributes which cannot be quantified otherwise was the statement attributed to Albert Einstein: "Not everything what counts can be counted, and not everything what can be counted – counts".

Appreciating your suggestion for numerical support of our research we carefully analyzed all survey questions in the questionnaire for the possibility of adding the additional quantitative questions as you had proposed. We realized then that only 6 of them, out of total 33 in the survey questionnaire could be improved in the way you suggested. Noticeably, these 6 questions constitute a rather small fraction of all 33 survey questions. Anyway, we decided to contact selected companies which formed our primary research sample and find out if they can help us to collect necessary data. We contacted 40 out of 74 previously selected companies operating within the confectionery industry in Poland and asked their representatives about quantifiable data concerning those eight questions which, in our opinion, could be numerically

supported. It occurred, that all of those companies, except 2 of them, do not collect data we were asking for. Then, we realized on this bases that 95% of all companies in the sample presumably could not provide any quantitative information we could use, thus its statistical examination in such a case would be pointless. So, we decided to search some statistics for the industry. Unfortunately, we also disappointed ourselves in this matter because there are no statistics for confectionery industry in Poland within the area of our interest. Although we found some statistics concerning the issue, they concerned the entire food industry in Poland what seemed for us ambiguous from the perspective of our examination. Eventually, we realized that the only solution we can provide to meet your suggestion to improve our research numerically is to present some statistics, as a secondary data, concerning sustainable performance of a few selected most recognizable international companies operating as well in the Poland's confectionery industry. The data together with our comment are introduced at the end of Methodological Section in lines 508-566 of the manuscript with marked changes, and in lines 506-564 of the unmarked revised manuscript.

We can only hope that our analysis built on those examples can be accepted as the case-based research that significantly improves our research numerically and, although partially, meet your suggestion to support our research with some quantitative data. Please, accept our apologies that we are unable to fully implement your proposition of our survey improvement.

Respectfully yours,

Pawel Tadeusz Kazibudzki, PhD
(on behalf of all authors)
